# Significado de los cuidadores informales sobre el cuidado de personas en situación de cronicidad

**DOI:** 10.1590/0034-7167-2025-0242es

**Published:** 2026-08-21

**Authors:** Alix Yaneth Perdomo-Romero, Claudia Patricia Cantillo-Medina, Claudia Andrea Ramírez-Perdomo

**Affiliations:** I Universidad Surcolombiana. Neiva, Huila, Colombia

**Keywords:** Cuidadores, Experiencias de Vida, Enfermedad Crónica, Dependencia, Investigación Cualitativa., Cuidadores, Experiências de Vida, Doença Crônica, Dependência, Pesquisa Qualitativa.

## Abstract

**Objetivos:**

comprender los significados que los cuidadores informales atribuyen al cuidado en su interacción con personas en situación de cronicidad.

**Métodos:**

estudio cualitativo, fenomenológico-hermenéutico. Participaron 20 cuidadores de personas con enfermedades crónicas y dependencia, utilizando la metodología de Van Manen.

**Resultados:**

emergieron cuatro temas: apoyo familiar como fuente de fortaleza emocional; espiritualidad como sustento del cuidado; complejidad económica del cuidado y lucha por el acceso a la atención en salud. Los cuidadores expresaron cómo el apoyo de sus familias y su fe les permiten sobrellevar la carga emocional, mientras que las dificultades económicas y las barreras en el acceso a la salud representan fuentes constantes de angustia.

**Conclusiones:**

los resultados demuestran la necesidad de políticas públicas que reconozcan el cuidado informal, mejoren las condiciones de los cuidadores, promuevan su bienestar físico y emocional a través de estrategias de apoyo financiero, formación y acceso equitativo a servicios de salud.

## RESUMO

**Objetivos:**

compreender os significados atribuídos ao cuidado por cuidadores informais na interação com pessoas com doenças crônicas.

**Métodos:**

estudo qualitativo, fenomenológico-hermenêutico. Vinte cuidadores de pessoas com doenças crônicas e dependência participaram, e a metodologia de Van Manen foi adotada.

**Resultados:**

quatro temas surgiram: o apoio familiar como fonte de força emocional; a espiritualidade como fonte de sustento do cuidado; a complexidade econômica do cuidado; e a luta pelo acesso aos cuidados de saúde. Os cuidadores expressaram que o apoio de suas famílias e sua fé lhes permitem lidar com o fardo emocional, enquanto as dificuldades econômicas e barreiras de acesso aos cuidados de saúde representam fontes constantes de angústia.

**Conclusões:**

os resultados demonstram a necessidade de políticas públicas que reconheçam os cuidados informais, melhorem as condições dos cuidadores e promovam seu bem-estar físico e emocional com estratégias de apoio financeiro, treinamento e acesso equitativo aos serviços de saúde.

## INTRODUCCIÓN

Se estima que para el año 2050^(1)^ más de dos mil millones de personas superarán los 60 años, lo que incrementará significativamente la prevalencia de enfermedades crónicas no transmisibles (ECNT) como el cáncer, la diabetes, las enfermedades cardiovasculares y respiratorias^(2)^. El envejecimiento acelerado en América Latina y el Caribe es un desafío porque afecta la disponibilidad de recursos humanos dedicados al cuidado de personas mayores con dependencia. Ante esta situación, se proyecta un incremento en la demanda de cuidadores profesionales, aproximadamente 9 millones para 2035 y más de 14 millones para 2050^(3)^.

El cuidador familiar es fundamental en la atención de personas con enfermedades crónicas y condiciones de dependencia a nivel mundial^(4)^. Ellos cuidan a familiares o personas cercanas que padecen enfermedades crónicas y/o discapacidades, sin recibir ningún tipo de remuneración o compensación por su labor^(3,5)^, con poco o ningún conocimiento para cuidar^(5-7)^. Este grupo representa el recurso humano más intensivo en términos de tiempo y esfuerzo, ya que, en muchos casos, se asume que dedican todo su tiempo al cuidado de una sola persona^(3)^. A pesar de los avances en los sistemas de salud, una parte significativa de los servicios de cuidado es provista por cuidadores informales^(3)^, pilares esenciales para garantizar el bienestar de las personas dependientes.

La labor de los cuidadores informales es indispensable, pero está marcada por desafíos que afectan su bienestar y la sostenibilidad de los sistemas de salud, especialmente en regiones de ingresos medios y bajos donde los recursos son limitados. En América Latina los cuidadores informales dedican más de 1.900 millones de horas al cuidado, representando entre el 0,26% y el 1,38% del PIB de los países de la región^(4)^. Este esfuerzo no remunerado, afecta la salud e impacta negativamente la empleabilidad de las mujeres, perpetuando la pobreza y la desigualdad de género^(8)^.

Culturalmente, el cuidado se considera una cualidad innata de las mujeres^(5,9-13)^. Datos de países como Chile, Colombia, Costa Rica, y México indican que entre el 63% y el 84% de los cuidadores no remunerados son mujeres, quienes asumen entre el 72% y el 88% del tiempo dedicado al cuidado^(10)^. Esta situación está profundamente ligada al fenómeno cultural del marianismo^(14)^, en el cual el género femenino es concebido como el grupo vulnerable que asume las labores del cuidado^(5,9,10,15,16)^.

El cuidado informal tiene importantes implicaciones físicas, emocionales y sociales^(5,9,17-21)^. Los cuidadores suelen enfrentar aislamiento social, agotamiento emocional, sobrecarga y un desequilibrio entre las necesidades de los demás y las propias^(20,22,23)^.

El apoyo familiar, desempeña un papel crucial como fuente de fortaleza emocional. La organización y colaboración dentro de las familias ayudan a mitigar el estrés y la carga asociados al cuidado^(12)^, mientras que el acompañamiento adecuado mejora la calidad de vida familiar. Además del apoyo familiar, muchos cuidadores encuentran en la espiritualidad un recurso fundamental para enfrentar los desafíos diarios^(6,12,20,24)^, ya que esta les ayuda a encontrar significado y resiliencia frente a las adversidades.

Por otro lado, los desafíos económicos del cuidado informal resaltan la complejidad de esta labor. La falta de compensación económica^(12)^ y la imposibilidad de mantener un empleo remunerado^(17,25)^, así como dificultades para acceder al mercado laboral^(17)^, generan inestabilidad financiera y dificultan la cobertura de gastos como la compra de medicamentos y tratamientos.

Además, los cuidadores enfrentan una constante lucha por atención en salud caracterizada por largos tiempos de espera, acceso limitado a terapias, demora en la entrega de medicamentos y recursos insuficientes^(12,26)^. Estas barreras erosionan la esperanza y confianza en los sistemas de salud, aumentando la carga emocional y física.

Desde una perspectiva disciplinar y humanística de la enfermería, se reconoce que el cuidado no puede comprenderse únicamente desde datos estadísticos, sino también a partir de las vivencias de quienes cuidan. En este sentido, la fenomenología hermenéutica permite aproximarse a la manera en que las personas otorgan significado a su experiencia, comprendiendo el acto de cuidar como una vivencia situada, cargada de sentidos, emociones y tensiones. Explorar esta realidad permite visibilizar las luchas y resiliencias del cuidador informal, y plantea interrogantes fundamentales sobre cómo los sistemas de salud y las políticas públicas pueden adaptarse para responder a estas realidades.

El interés por estudiar estas experiencias surge, de la necesidad de comprender cómo los cuidadores viven, sienten y resignifican el cuidado en contextos de cronicidad, donde convergen dimensiones emocionales, espirituales, económicas y sociales, integrando estos aspectos a la investigación y a la práctica profesional como un paso esencial hacia un enfoque inclusivo que garantice su bienestar y el de quienes dependen de ellos.

## OBJETIVOS

Comprender los significados que los cuidadores informales atribuyen al cuidado en su interacción con personas en situación de cronicidad.

## MÉTODOS

### Aspectos éticos

El estudio fue aprobado por el Comité de Ética y Bioética. Se respetaron los principios éticos de beneficencia, no maleficencia, autonomía y justicia. Para proteger la privacidad de los participantes, se utilizó un sistema de codificación del C1 al C20, en lugar de nombres reales en la transcripción y el análisis de datos.

Todos los participantes firmaron el consentimiento informado antes de iniciar su participación en el estudio.

Se llevó a cabo un estudio cualitativo, bajo un enfoque fenomenológico-hermenéutico, fundamentado en la metodología propuesta por Van Manen^(27)^. Este enfoque permitió explorar y comprender los significados atribuidos al cuidado por los cuidadores informales en su interacción con personas en situación de cronicidad. La fenomenología hermenéutica se eligió debido a su capacidad para captar las experiencias subjetivas y los significados profundos que los participantes otorgan a su labor, integrando tanto una dimensión descriptiva como interpretativa^(28)^. Este diseño fue seleccionado porque permite no solo describir las experiencias vividas, sino también interpretar cómo estas son comprendidas y comunicadas por los cuidadores. Este estudio siguió las directrices del *Consolidated Qualitative Research Reporting Criteria* (COREQ) para estudios cualitativos^(28)^.

### Entorno de estudio

El estudio se llevó a cabo en el municipio de Neiva, departamento del Huila, al sur de Colombia, con los cuidadores que participan en el programa orientado al apoyo de cuidadores, lo que facilitó la identificación y selección de los participantes.

### Fuente de datos

Se empleó un muestreo casual no probabilístico orientado por criterio, para seleccionar a los cuidadores participantes, vinculados al programa Soporte al Cuidado y al Cuidador, liderado por el programa de Enfermería de la Universidad Surcolombiana. Este tipo de muestreo permitió garantizar la profundidad y la calidad de los datos recopilados^(29)^, asegurando la riqueza narrativa. La selección de participantes se orientó a obtener testimonios que aportaran información y que reflejaran la diversidad de experiencias y percepciones del cuidado, permitiendo recuperar la riqueza de la experiencia vivida.

El programa Soporte al Cuidado y al Cuidador contaba, al momento del estudio, con aproximadamente 47 cuidadores activos. De este total, 42 cuidadores cumplieron los criterios de inclusión y, 5 cuidadores fueron excluidos, principalmente por encontrarse cuidando personas en fase aguda de la enfermedad. Finalmente, 20 cuidadores conformaron el grupo de participantes, número considerado suficiente para el abordaje fenomenológico-hermenéutico del fenómeno estudiado.

Los criterios de inclusión fueron ser mayor de 18 años, haber ejercido como cuidador por al menos tres meses y dedicar más de tres horas diarias al cuidado. Se excluyeron cuidadores de personas con enfermedad en crisis aguda.

### Recopilación y organización de datos

La recolección de datos se realizó entre marzo de 2022 y junio de 2023. Los cuidadores fueron contactados a través del programa Soporte al Cuidado y al Cuidador durante las actividades presenciales de acompañamiento y seguimiento realizadas por el Programa de Enfermería. No se registraron retiros durante el proceso de recolección de la información. El número final de 20 participantes se alcanzó tras constatar la suficiencia de información para la construcción de textos fenomenológicos enriquecidos. Se llevaron a cabo entrevistas en profundidad^(27)^, realizadas por la investigadora principal quien contaba con experiencia en investigación fenomenológica, en espacios previamente acordados con cada participante para garantizar la privacidad y comodidad. La pregunta de apertura fue ¿Cuénteme qué ha significado para usted la experiencia de ser cuidador informal? Esta pregunta abierta permitió explorar las percepciones, emociones y significados atribuidos al cuidado desde una perspectiva personal. Cada entrevista tuvo una duración promedio de 60 minutos y fue grabada con el consentimiento previo del participante.

Las grabaciones fueron transcritas en un plazo máximo de 24 horas por auxiliares capacitados, utilizando un enfoque semántico para preservar la fidelidad del discurso original. Se garantizaron la seguridad y la confidencialidad de las grabaciones, las cuales fueron almacenadas y custodiadas por las investigadoras.

Para asegurar la calidad metodológica, se aplicaron los criterios propuestos por Lincoln y Guba^(30)^. La credibilidad y la conformabilidad se garantizaron mediante la devolución de las entrevistas a los participantes para confirmar si se sentían identificados con los textos; la transferibilidad fue asegurada a través de la descripción detallada del contexto y los hallazgos; y la confiabilidad a través de la claridad metodológica.

### Análisis de datos

El análisis se desarrolló siguiendo los pasos metodológicos propuestos por Van Manen^(27)^: transcripción y lectura reflexiva, las entrevistas fueron leídas repetidamente para familiarizarse con el contenido; el análisis semántico permitió identificar palabras claves, frases significativas y patrones recurrentes en las narrativas; llevando a la identificación de temas emergentes y la articulación de patrones de significado, interpretados desde una perspectiva hermenéutica, buscando comprender cómo los cuidadores atribuyen significado a sus experiencias.

El proceso de análisis se desarrolló de manera progresiva, cíclica e interpretativa, avanzando de la comprensión global a la particular y retornando constantemente a la globalidad, en coherencia con la fenomenología hermenéutica propuesta por Van Manen^(27)^. Durante el proceso investigativo se asumió la epoché como una actitud reflexiva orientada a la suspensión de juicios previos sobre el mundo natural^(31)^, favoreciendo una apertura comprensiva frente a las experiencias narradas por los cuidadores y permitiendo que el significado emergiera desde la propia vivencia^(27)^.

Inicialmente, se realizó una lectura global de las transcripciones con el fin de obtener una comprensión general de las experiencias relatadas. Posteriormente, se efectuaron lecturas detalladas, orientadas a identificar expresiones significativas vinculadas al fenómeno del cuidado. A partir de este proceso, se delimitaron unidades de significado, las cuales fueron contrastadas entre las entrevistas para reconocer patrones comunes e interconexiones. Estos patrones dieron lugar a la construcción de temas fenomenológicos, desarrollados mediante un ejercicio continuo de escritura y reescritura reflexiva, para analizar e interpretar el significado de la experiencia vivida.

De forma paralela se realizaron encuentros grupales entre las investigadoras, en los cuales se discutieron los resultados hasta alcanzar un consensos interpretativos y definir los temas emergentes, mediante un proceso de triangulación de investigadores^(32)^.

## RESULTADOS

Se entrevistaron a 20 cuidadores, quienes brindaban atención a familiares cercanos como esposos(as), hijos, madres o padres; de los cuales doce eran mujeres y ocho hombres, con una edad media de 51 años. Los cuidadores se encargaban de atender a familiares con diversos diagnósticos, entre ellos enfermedad renal crónica, secuelas de COVID-19, problemas de salud mental, Alzheimer y discapacidades cognitivas y musculares.

Del análisis de las entrevistas emergieron cuatro temas principales que reflejan las experiencias y desafíos enfrentados por los cuidadores.

### El apoyo familiar como fuente de fortaleza emocional.

Los cuidadores destacan la importancia de la red familiar en el proceso de cuidado. Este apoyo se manifiesta en diversas áreas, incluido el cuidado físico, emocional, social y económico. El apoyo familiar permite a los cuidadores afrontar las dificultades del cuidado con mayor fortaleza. Con el tiempo la familia logra organizarse de tal forma que trabaja en equipo para cuidar a su ser querido, compartiendo la responsabilidad, aunque siempre hay uno de ellos que guía y lidera el proceso de cuidado.

*Mi señora es muy puntual en eso y ha sido un apoyo fundamental* [...] *porque en estos momentos mi esposa es la que la baña y la ayuda a vestir* [...]. (C1)

*Si, ellos son el apoyo de nosotros, ellos están muy pendientes de ella.* (C2)

*Cuando tengo alguna vuelta por hacer* [...]*, pues la dejo con mi mamá.* (C3)

*Mi mamá es un apoyo fundamental, si no fuera por ella no podría con todo esto.* (C15)

*Verlo así me duele, afortunadamente mis hijos viven pendientes y me ayudan, ellos son mi fortaleza.* (014)

*Mis hermanos se turnan conmigo, eso me ayuda a sobrellevar todo esto.* (C10)

### La espiritualidad como sustento del cuidado.

La espiritualidad y la fe en un ser superior son fundamentales para mantener una actitud positiva y resiliente frente a situaciones desafiantes. Los cuidadores mencionan que su creencia en Dios les proporciona consuelo y esperanza frente a las adversidades del cuidado. Esta dimensión espiritual les ayuda a encontrar significado en su labor diaria.

*Yo creo que es la fe, creer en un Dios ayuda para uno confiar* [...]*, uno va asumiendo esas situaciones y a medida que se van presentando va dando la solución.* (C2)

*Uno ante todo tiene fe* [...]*, la fe nunca se pierde, hay que ser realista, hay que tener la posibilidad de que Dios se lo sane a uno.* (C5)

*Dios no pone cargas que uno no pueda llevar* [...]*, esta carga ha sido muy dura.* (C17)

*Yo le pido mucho a Dios, que me ayude, a darme salud, para cuidarlo a él.* (C18)

*Dios me da fuerzas para no rendirme.* (C11)

*La oración me sostiene cuando siento que ya no puedo más.* (C13)

### La complejidad económica del cuidado.

Las expresiones reflejan directamente las dificultades económicas y laborales que enfrentan al asumir el rol de cuidadores. La imposibilidad de mantener un empleo remunerado debido a las demandas del cuidado los lleva a renunciar, lo que da como resultado la pérdida de ingresos y estabilidad económica. Los cuidadores enfrentan desafíos emocionales al equilibrar las responsabilidades laborales y familiares. La decisión de renunciar a sus trabajos muestra los sacrificios que están dispuestos a hacer para priorizar el cuidado de su ser querido

*Entonces todo eso se le complica a uno de cuidador con la persona, porque, aunque uno la pudiera dejar sentada y ella se incorpore, pues sale uno a trabajar* [...], *pero es super, complicado que dependa de uno, no puedo salir a trabajar, entonces eso es duro.* (C4)

*Que uno no pueda tener un trabajo* [...], *si yo consigo trabajo quién me cuida mi hija, yo quisiera trabajar para poder aportar más, pero no lo puedo hacer.* (C3)

*Me tocó dejar mi taller a un lado y dedicarme el cien por ciento a él* [...]. *La preocupación ¿qué voy a hacer? ¿Cómo le voy a dar las cosas si no tengo esto si no tengo lo otro?* (C6)

*He renunciado a muchos trabajos por ella* [...]*, me tocaba decir no puedo laborar más, mi mujer está enferma. Me tocó renunciar y si me dolió.* (C8)

*Me tocó dejar de trabajar porque ella me necesita todo el tiempo.* (C12)

### La lucha por el acceso a la atención en salud.

Los cuidadores enfrentan barreras significativas al intentar acceder a servicios de salud para sus familiares, esto los lleva a experimentar desesperanza ante un sistema de salud deficiente, que obstaculiza el cuidado y que incluye largos tiempos de espera, falta de acceso a terapias y medicamentos, y una atención deficiente por parte del personal médico y de enfermería. En la mayoría de las oportunidades deben recurrir a demandas contra el sistema de salud. A continuación, se presentan algunas de las expresiones de los participantes:

*Se complica la situación también de las citas, son muy largas* [...], *me la dieron en mayo y lleva esperando tres meses para que la atiendan, entonces ese proceso es complicado*. (C4)

*La atención en la clínica es pésima*. (C7)

*El cuidado formal solo es posible cuando están demasiado complicados, como si tener dependencia total no fuera ya difícil, todo el sistema está en contra nuestra, ¿quién nos ayuda o entiende?, nadie, absolutamente nadie.* (C6)

*Cuando los trasladan al piso el cuidador debe bañarlo, cambiarlo de posición, cambiarle el pañal, darle comida, los auxiliares tratan mal al cuidador. Responden de manera displicente que es responsabilidad de los cuidadores hacer todos los procedimientos, no se ellos para qué están destinados o cuáles son sus funciones.* (C9)

*Nos mandan de un lado a otro y nadie asume la responsabilidad*. (C19)

*A veces no hay el especialista que requiere mi hijo, y cuando al fin se logra la cita, le dicen a uno que vuelva en seis meses.* (C16)

*Ya perdí la cuenta de cuántas veces he tenido que demandar al sistema de salud.* (C20)

## DISCUSIÓN

Los hallazgos reflejan la complejidad del papel del cuidador informal, donde los aspectos sociales, familiares, económicos y de los sistemas de salud influyen significativamente en su experiencia. La discusión se articulará en torno a los temas emergentes identificados en el grupo de cuidadores quienes se caracterizan por ser en su mayoría mujeres, con vinculo familiar con las persons cuidadas, con edades por encima de los 50 años principalmente.

El apoyo familiar surge como un pilar esencial para los cuidadores informales, proporcionando respaldo emocional y práctico que mitiga la carga del cuidado. La colaboración dentro del entorno familiar no solo reduce los niveles de estrés, sino que también mejora la calidad de vida de los cuidadores, permitiéndoles encontrar un equilibrio entre sus responsabilidades y necesidades personales. Este hallazgo coincide con estudios que destacan la importancia del apoyo social y familiar en el bienestar de los cuidadores^(7,21,33)^.

Por otro lado, la espiritualidad actúa como un recurso resiliente que permite a los cuidadores enfrentar las adversidades diarias, encontrar significado en su labor y mantener la esperanza frente a situaciones desafiantes. Este hallazgo está respaldado por investigaciones que evidencian cómo la fe y las prácticas espirituales actúan como mecanismos efectivos de afrontamiento para los cuidadores^(34)^. Además, la espiritualidad fomenta una conexión profunda con los seres queridos, proporcionando un marco para entender y aceptar las situaciones difíciles del cuidado.

Las dificultades económicas representan otro desafío significativo, ya que muchos cuidadores deben abandonar sus empleos remunerados^(15)^, lo cual conduce a una dependencia económica^(35)^.

Las mujeres desempeñan un papel predominante en el cuidado no remunerado y enfrentan una carga significativa sin recibir compensación económica^(15,36)^, una responsabilidad que el sistema actual no logra garantizar ni equilibrar adecuadamente. Estas situaciones económicas complejas hacen necesario abordar y mitigar los desafíos financieros que enfrentan los cuidadores, así como reconocer y valorar su labor dentro de la estructura social y económica.

Es importante que el Estado, la sociedad y la familia revisen y aborden esta desigualdad estructural^(36)^. Además, es fundamental considerar el cuidado como un derecho universal, que promueve el bienestar humano y constituye un elemento central en el diseño de políticas públicas^(37)^. Por consiguiente, es crucial implementar políticas que brinden apoyo financiero, como subsidios económicos y programas de capacitación, para aliviar la carga económica y mejorar la calidad de vida de los cuidadores.

Igualmente, el acceso a la atención en salud se configura como una batalla agotadora y desafiante para garantizar que sus seres queridos reciban la atención en salud que necesitan^(38)^. Los cuidadores se ven atrapados en un laberinto burocrático y enfrentan numerosos obstáculos para acceder a servicios médicos y de atención adecuados, por lo que recurren a demandas judiciales para lograr ser escuchados y obtener lo que tienen derecho. Esta lucha constante quebranta su esperanza y confianza, dejándolos desanimados y desalentados ante la aparente inaccesibilidad de la atención en salud para aquellos a quienes cuidan.

A medida que enfrentan una serie de dificultades, desde largas listas de espera hasta barreras financieras y administrativas, la esperanza de los cuidadores se desvanece, pues luchan no solo por el bienestar de sus seres queridos, sino también por su propia salud mental y emocional. Este hallazgo demuestra la necesidad urgente de reformar los sistemas de atención en salud para garantizar que tanto los cuidadores como las personas con dependencia asociada a la enfermedad reciban el apoyo necesario.

En este sentido, la experiencia del cuidar se configura como una vivencia que atraviesa y tensiona la vida familiar, la economía, la espiritualidad y la relación con el sistema de salud, dando lugar a un mundo marcado por la resistencia silenciosa, la entrega cotidiana y el desgaste continuo. La vida del cuidador se reorganiza profundamente: la familia resulta esencial, la fe sostiene, la esperanza se fractura y el sistema pone a prueba constantemente la capacidad de resistencia del cuidador. Así, el cuidado se vive como una experiencia compleja y profundamente humana, que transforma inexorablemente la forma de vivir.

Este estudio contribuye a visibilizar las luchas diarias de los cuidadores informales y plantea interrogantes sobre cómo los sistemas de salud, económicos y sociales pueden abordar y responder a estas realidades. Integrar estos aspectos en futuras investigaciones permitirá desarrollar estrategias efectivas que reconozcan y respalden la labor invaluable de los cuidadores informales, garantizando su bienestar y el de las personas a quienes cuidan.

### Limitaciones del estudio

Este estudio se desarrolló en un contexto social y geográfico específico, por lo que las experiencias analizadas reflejan las vivencias de los cuidadores participantes en ese escenario particular.

### Contribuciones a la enfermería, la salud o las políticas públicas

Este estudio contribuye a la generación de nuevo conocimiento en enfermería al visibilizar la experiencia de los cuidadores informales desde una perspectiva fenomenológica-hermenéutica, destacando dimensiones esenciales como el apoyo familiar, la espiritualidad, las barreras económicas y el acceso a la salud. Los hallazgos permiten ampliar la comprensión del impacto del cuidado informal en la calidad de vida de los cuidadores y evidencian la necesidad de intervenciones que fortalezcan su bienestar. Además, fundamentan la creación de políticas públicas e intervenciones en enfermería que promuevan el bienestar mediante un acompañamiento integral, fomentando la humanización del cuidado y la corresponsabilidad en los sistemas de salud.

## CONSIDERACIONES FINALES

El crecimiento exponencial de las necesidades de recursos humanos para el cuidado pone de manifiesto la urgencia de implementar políticas públicas que fortalezcan los sistemas de atención. Este estudio pone de manifiesto el difícil papel de los cuidadores informales, un rol fundamental para el bienestar de las personas con enfermedades crónicas y/o dependientes.

Los hallazgos destacan cómo el apoyo familiar no solo actúa como un recurso esencial para mitigar el estrés asociado al cuidado, sino que también contribuye a la resiliencia emocional de los cuidadores. Sin embargo, las barreras económicas y el acceso limitado a los servicios de salud representan desafíos significativos que requieren atención urgente.

Es imperativo que las políticas públicas reconozcan y valoren el trabajo de los cuidadores informales e implementen estrategias que mejoren sus condiciones laborales y promuevan su bienestar físico y emocional. Esto incluye el desarrollo de programas de formación, apoyo financiero, así como la creación de redes de apoyo que mitiguen la sobrecarga y fomenten una distribución más equitativa de las responsabilidades de cuidado. Estas acciones son fundamentales para garantizar una atención digna y sostenible, mejorar la calidad de vida de los cuidadores y promover su bienestar integral en la región.

## Data Availability

Los datos de la investigación están disponibles en el cuerpo del artículo.
