# Supplementary material for: Significado de los cuidadores informales sobre el cuidado de personas en situación de cronicidad
Source: Rev Bras Enferm. 2026 Aug 21;79:e20250242. [Article in Spanish] doi: 10.1590/0034-7167-2025-0242es (PMC13505555; doi:10.1590/0034-7167-2025-0242es)
Supplement: Translation

# Translation: Meaning of informal caregivers in the care of people with chronic conditions

**DOI:** 10.1590/0034-7167-2025-0242

**Authors:** Alix Yaneth Perdomo-Romero, Claudia Patricia Cantillo-Medina, Claudia Andrea Ramírez-Perdomo

**Affiliations:** I Universidad Surcolombiana. Neiva, Huila, Colombia

**Keywords:** Caregivers, Life Experiences, Chronic Illness, Dependency, Qualitative Research.

## Abstract

**Objectives:**

to understand the meanings attributed to caregiving by informal caregivers in their interaction with people with chronic conditions.

**Methods:**

qualitative, phenomenological-hermeneutic study. Twenty caregivers of people with chronic illnesses and dependency participated, using Van Manen’s methodology.

**Results:**

four themes emerged: family support as a source of emotional strength; spirituality as a source of support for caregiving; the economic complexity of caregiving; and the struggle for access to health care. Caregivers expressed how the support of their families and their faith enable them to cope with the emotional burden, while economic difficulties and barriers to accessing health care represent constant sources of distress.

**Conclusions:**

the results demonstrate the need for public policies that recognize informal caregiving, improve caregivers’ conditions, and promote their physical and emotional well-being through financial support strategies, training, and equitable access to health services.

## INTRODUCTION

By 2050^(1)^, more than two billion people are expected to be over the age of 60, which will significantly increase the prevalence of chronic noncommunicable diseases (CNCDs) such as cancer, diabetes, and cardiovascular and respiratory diseases^(2)^. Accelerated aging in Latin America and the Caribbean is a challenge because it affects the availability of human resources dedicated to caring for dependent older adults. Given this situation, an increase in the demand for professional caregivers is projected: approximately 9 million by 2035 and more than 14 million by 2050^(3)^.

Family caregivers play a vital role in caring for people with chronic illnesses and dependency conditions worldwide^(4)^. They care for family members or close friends who suffer from chronic illnesses and/or disabilities, without receiving any remuneration or compensation for their work^(3,5)^, with little or no knowledge of how to care for^(5-7)^. This group represents the most timeand effort-intensive human resource, since in many cases it is assumed that they devote all their time to caring for a single person^(3)^. Despite advances in health systems, a significant portion of care services is provided by informal caregivers^(3)^, who are essential pillars in ensuring the well-being of dependent persons.

The work of informal caregivers is indispensable, but it is marked by challenges that affect their well-being and the sustainability of health systems, especially in lowand middle-income regions where resources are limited. In Latin America, informal caregivers devote more than 1.9 billion hours to caregiving, representing between 0.26% and 1.38% of the GDP of countries in the region^(4)^. This unpaid work affects women’s health and negatively impacts their employability, perpetuating poverty and gender inequality^(8)^.

Culturally, caregiving is considered an innate quality of women^(5,9-13)^. Data from countries such as Chile, Colombia, Costa Rica, and Mexico indicate that between 63% and 84% of unpaid caregivers are women, who account for between 72% and 88% of the time devoted to caregiving^(10)^. This situation is deeply linked to the cultural phenomenon of Marianism^(14)^, in which women are a vulnerable group that takes on caregiving tasks^(5,9,10,15,16)^.

Informal care has significant physical, emotional, and social implications^(5,9,17-21)^. Caregivers often face social isolation, emotional exhaustion, overload, and an imbalance between the needs of others and their own^(20,22,23)^.

Family support plays a crucial role as a source of emotional strength. Organization and collaboration within families help mitigate the stress and burden associated with caregiving^(12)^, whereas appropriate support improves the quality of family life. In addition to family support, many caregivers find spirituality to be a fundamental resource for coping with daily challenges^(6,12,20,24)^, which helps them find meaning and resilience in the face of adversity.

On the other hand, the economic challenges of informal caregiving highlight the complexity of this work. The lack of financial compensation^(12)^ and the inability to hold down a paid job^(17,25)^, as well as difficulties accessing the labor market^(17)^, create financial instability and make it difficult to cover expenses such as the purchase of medicines and treatments. In addition, caregivers face a constant struggle for health care characterized by long wait times, limited access to therapies, delays in medication delivery, and insufficient resources^(12,26)^. These barriers erode hope and trust in healthcare systems, increasing the emotional and physical burden.

From a disciplinary and humanistic perspective of nursing, it is recognized that care cannot be understood solely from statistical data, but rather from the experiences of those who provide care. In this sense, hermeneutic phenomenology allows us to approach the way people give meaning to their experience, understanding the act of caring as a situated experience, charged with meaning, emotions, and tensions. Exploring this reality allows us to visualize the struggles and resilience of informal caregivers and raises fundamental questions about how health systems and public policies can adapt to respond to these realities.

The interest in studying these experiences arises from the need to understand how caregivers live, feel, and reframe caregiving in chronic contexts, where emotional, spiritual, economic, and social dimensions converge, integrating these aspects into research and professional practice as an essential step toward an inclusive approach that guarantees their well-being and that of those who depend on them.

## OBJECTIVES

To understand the meanings attributed to caregiving by informal caregivers in their interaction with people with chronic conditions.

## METHODS

### Ethical considerations

The study was approved by the Ethics and Bioethics Committee. The ethical principles of beneficence, non-maleficence, autonomy, and justice were respected. To protect participants’ privacy, a coding system from C1 to C20 was used instead of real names in the transcription and data analysis. All participants signed an informed consent form before participating in the study.

A qualitative study was conducted using a phenomenological-hermeneutic approach based on the methodology proposed by Van Manen^(27)^. This approach allowed us to explore and understand the meanings attributed to caregiving by informal caregivers in their interaction with people with chronic conditions. Hermeneutic phenomenology was chosen because of its ability to capture the subjective experiences and deep meanings that participants attach to their work, integrating both descriptive and interpretive dimensions^(28)^. This design was selected because it allows not only for the description of lived experiences, but also for the interpretation of how these are understood and communicated by caregivers. This study followed the guidelines of the Consolidated Criteria for Reporting Qualitative Research (COREQ)^(28)^ for qualitative studies.

### Study setting

The study was conducted in the municipality of Neiva, Huila department, southern Colombia, with caregivers participating in a caregiver support program, which facilitated the identification and selection of participants.

### Data source

A non-probabilistic, criterion-guided random sampling was used to select the participating caregivers, linked to the Care and Caregiver Support program, led by the Nursing Program at the Universidad Surcolombiana. This type of sampling ensured the depth and quality of the collected data^(29)^, thereby enhancing narrative richness. The selection of participants was aimed at obtaining testimonies that would provide information and reflect the diversity of experiences and perceptions of care, allowing the richness of the lived experience to be recovered.

At the time of the study, the Care and Caregiver Support program had approximately 47 active caregivers. Of this total, 42 caregivers met the inclusion criteria, while 5 caregivers were excluded, mainly because they were caring for people in the acute phase of the disease. Finally, 20 caregivers made up the group of participants, a number considered sufficient for the phenomenological-hermeneutic approach to the phenomenon under study.

The inclusion criteria were being over 18 years of age, having been a caregiver for at least three months, and dedicating more than three hours a day to caregiving. Caregivers of people in the acute phase of the disease were excluded.

### Data collection and organization

Data collection took place between March 2022 and June 2023. Caregivers were contacted through the Care and Caregiver Support program during face-to-face support and follow-up activities carried out by the Nursing Program. No withdrawals were recorded during the information collection process. The final number of 20 participants was reached after verifying that there was sufficient information to construct enriched phenomenological texts. In-depth interviews were conducted^(27)^ by the main researcher, who had experience in phenomenological research, in spaces previously agreed upon with each participant to ensure privacy and comfort. The opening question was: “What does the experience of being an informal caregiver mean to you?”. This open-ended question allowed for the exploration of perceptions, emotions, and meanings attributed to caregiving from a personal perspective. Each interview lasted an average of 60 minutes and was recorded with the participant’s prior consent.

The recordings were transcribed within 24 hours by trained assistants, using a semantic approach to preserve the fidelity of the original discourse. The security and confidentiality of the recordings were guaranteed, and they were stored and safeguarded by the researchers.

To ensure methodological quality, the criteria proposed by Lincoln and Guba were applied^(30)^. Credibility and conformability were ensured by returning the interviews to the participants to confirm whether they identified with the texts; transferability was ensured through a detailed description of the context and findings; and reliability was guaranteed through methodological clarity.

### Data analysis

The analysis was conducted following the methodological steps proposed by Van Manen^(27)^: transcription and reflective reading, the interviews were read repeatedly to familiarize ourselves with the content; semantic analysis allowed us to identify keywords, significant phrases, and recurring patterns in the narratives; leading to the identification of emerging themes and the articulation of patterns of meaning, interpreted from a hermeneutic perspective, seeking to understand how caregivers attribute meaning to their experiences.

The analysis process was carried out in a progressive, cyclical, and interpretive manner, moving from a global understanding to a particular one and constantly returning to the global, in line with the hermeneutic phenomenology proposed by Van Manen^(27)^. During the research process, epoché was adopted as a reflective attitude aimed at suspending prior judgments about the natural world^(31)^, fostering comprehensive openness to the experiences narrated by caregivers and allowing meaning to emerge from the experience itself^(27)^.

Initially, a comprehensive reading of the transcripts was conducted in order to gain an understanding of the experiences recounted. Subsequently, detailed readings were carried out, aimed at identifying significant expressions related to the phenomenon of caregiving. Based on this process, units of meaning were identified and compared across interviews to recognize common patterns and interconnections. These patterns gave rise to the construction of phenomenological themes, developed through a continuous exercise of reflective writing and rewriting, to analyze and interpret the meaning of the lived experience.

At the same time, group meetings were held among the researchers, in which the results were discussed until an interpretative consensus was reached and emerging themes were defined, through a process of researcher triangulation^(32)^.

## RESULTS

Twenty caregivers were interviewed, all of whom provided care to close relatives such as spouses, children, or parents. Twelve were women and eight were men, with an average age of 51. The caregivers were responsible for caring for family members with various diagnoses, including chronic kidney disease, COVID-19 sequelae, mental health problems, Alzheimer’s, and cognitive and muscular disabilities.

Four main themes emerged from the analysis of the interviews, reflecting the experiences and challenges caregivers face.

### Family support as a source of emotional strength

Caregivers emphasize the importance of the family network in the caregiving process. This support manifests itself in various areas, including physical, emotional, social, and financial care. Family support enables caregivers to cope with the difficulties of caregiving with greater strength. Over time, the family manages to organize itself so they work as a team to care for their loved one, sharing responsibility, though one of them always guides and leads the caregiving process.

*My wife is very punctual in this regard and has been a fundamental support* [...] *because right now my wife is the one who bathes her and helps her get dressed* [...]. (C1)

*Yes, they are our support, they are very attentive to her.* (C2)

*When I have something to do* [...]*, I leave her with my mom.* (C3)

*My mom is a fundamental support, if it weren’t for her, I couldn’t cope with all this.* (C15)

*Seeing him like this hurts me, fortunately my children are attentive and help me, they are my strength.* (C14)

*My siblings take turns with me, that helps me cope with all this*. (C10)

### Spirituality as a source of support for caregiving

Spirituality and faith in a higher power are fundamental to maintaining a positive and resilient attitude in the face of challenging situations. Caregivers note that their belief in God provides them with comfort and hope amid the adversities of caregiving. This spiritual dimension helps them find meaning in their daily work.

*I believe that faith, believing in God, helps you to trust* [...]*, you accept these situations and as they arise, you find the solution.* (C2)

*First and foremost, you have faith* [...]*, faith is never lost, you have to be realistic, you have to believe that God can heal you.* (C5)

*God does not give us burdens that we cannot bear* [...]*, this burden has been very hard.* (C17)

*I ask God a lot to help me, to give me health, so that I can take care of him.* (C18)

*God gives me the strength not to give up.* (C11)

*Prayer sustains me when I feel I can’t go on any longer.* (C13)

### The economic complexity of caregiving

These expressions directly reflect the economic and employment difficulties they face when taking on the role of caregivers. The inability to maintain paid employment due to caregiving demands leads them to resign, resulting in loss of income and economic stability. Caregivers face emotional challenges in balancing work and family responsibilities. The decision to resign from their jobs shows the sacrifices they are willing to make to prioritize the care of their loved one.

*So all of that complicates things for the caregiver, because even though you can leave her sitting there and she can sit up by herself, you have to go to work* [...]*, but it’s really complicated when she depends on you. I can’t go out to work, so that’s hard.* (C4)

*Not being able to have a job* [...]*, if I get a job, who will take care of my daughter? I would like to work to be able to contribute more, but I can’t do it*. (C3)

*I had to put my workshop aside and devote myself 100% to him* [...]*. The worry of what am I going to do? How am I going to provide for her if I don’t have this or that?* (C6)

*I’ve given up many jobs because of her* [...]*, I had to say I can’t work anymore, my wife is sick. I had to quit and it hurt.* (C8)

*I had to stop working because she needs me all the time.* (C12)

### The struggle for access to health care

Caregivers face significant barriers when trying to access health services for their family members, leading them to feel hopeless in the face of a deficient health system that hinders care and includes long waiting times, lack of access to therapies and medications, and poor care from medical and nursing staff. In most cases, they must file complaints with the health system. Below are some of the participants’ comments:

*The situation with appointments is also complicated; they take a long time* [...]*, I was given one in May and have been waiting three months to be seen, so that process is complicated.* (C4)

*The care at the clinic is terrible.* (C7)

*Formal care is only possible when things are too complicated, as if being totally dependent wasn’t already difficult enough. The whole system is against us. Who helps us or understands us? No one, absolutely no one.* (C6)

*When they are transferred to the floor, the caregiver must bathe them, change their position, change their diaper, feed them, and the assistants treat the caregiver badly. They respond dismissively that it is the caregivers’ responsibility to do all the procedures. I don’t know what they are for or what their functions are.* (C9)

*They send us from one place to another and no one takes responsibility.* (C19)

*Sometimes there is no specialist available for my child, and when we finally get an appointment, they tell us to come back in six months.* (C16)

*I’ve lost count of how many times I’ve had to sue the healthcare system.* (C20)

## DISCUSSION

The findings reflect the complexity of the informal caregiver role, in which social, family, economic, and health system factors significantly influence their experience. The discussion will focus on the emerging themes identified in the group of caregivers, who are mostly women, with family ties to the caregivers, and mainly over the age of 50.

Family support emerges as an essential pillar for informal caregivers, providing emotional and practical support that mitigates the burden of care. Collaboration within the family environment not only reduces stress levels but also improves caregivers’ quality of life, enabling them to balance their responsibilities and personal needs. This finding agrees with studies that highlight the importance of social and family support for caregivers’ well-being^(7,21,33)^.

On the other hand, spirituality serves as a resilient resource, helping caregivers face daily adversities, find meaning in their work, and maintain hope in the face of challenging situations. This finding is supported by research showing that faith and spiritual practices act as effective coping mechanisms for caregivers^(34)^. In addition, spirituality fosters a deep connection with loved ones, providing a framework for understanding and accepting difficult caregiving situations.

Financial difficulties represent another significant challenge, with many caregivers having to give up their paid jobs^(15)^, which leads to economic dependence^(35)^.

Women play a predominant role in unpaid care work and face a significant burden without receiving financial compensation^(15,36)^, a responsibility that the current system fails to adequately guarantee or balance. These complex economic situations require addressing and mitigating caregivers’ financial challenges, as well as recognizing and valuing their work within the social and economic structure.

It is important that the state, society, and families review and address this structural inequality^(36)^. Furthermore, it is essential to consider care as a universal right that promotes human well-being and is a central element in the design of public policies^(37)^. Therefore, it is crucial to implement policies that provide financial support, such as economic subsidies and training programs, to alleviate the economic burden on caregivers and improve their quality of life.

Similarly, access to health care is an exhausting and challenging battle to ensure that their loved ones receive the care they need^(38)^. Caught in a bureaucratic maze and facing numerous obstacles to accessing adequate medical and care services, caregivers resort to legal action to make themselves heard and obtain what they are entitled to. This constant struggle undermines their hope and confidence, leaving them discouraged and disheartened by the apparent inaccessibility of health care for those they care for.

As they face a series of difficulties, from long waiting lists to financial and administrative barriers, caregivers’ hope fades, and they struggle not only for the well-being of their loved ones, but also for their own mental and emotional health. This finding demonstrates the urgent need to reform healthcare systems to ensure that both caregivers and people with illness-related dependency receive the necessary support.

In this sense, caregiving is shaped as an experience that permeates and strains family life, finances, spirituality, and the relationship with the healthcare system, giving rise to a world marked by silent resistance, daily dedication, and continuous exhaustion. Caregivers’ lives are profoundly reorganized: the family is essential, faith sustains, hope is fractured, and the system constantly tests their ability to resist. Thus, caregiving is experienced as a complex and deeply human endeavor that inexorably transforms the way of living.

This study highlights the daily struggles of informal caregivers and raises questions about how health, economic, and social systems can address these realities. Integrating these aspects into future research will enable the development of effective strategies that recognize and support the invaluable work of informal caregivers, ensuring their well-being and that of the people they care for.

### Study limitations

This study was conducted in a specific social and geographical context, so the experiences included reflect those of the caregivers participating in that setting.

### Contributions to nursing, health, or public policy

This study contributes new knowledge to nursing by highlighting the experiences of informal caregivers from a phenomenological-hermeneutic perspective, emphasizing essential dimensions such as family support, spirituality, economic barriers, and access to health care. The findings broaden our understanding of the impact of informal care on caregivers’ quality of life, highlighting the need for interventions that strengthen their well-being. Furthermore, it provides a basis for creating public policies and nursing interventions that promote well-being through comprehensive support, fostering the humanization of care and shared responsibility within health systems.

## FINAL CONSIDERATIONS

The exponential growth in human resource needs for care highlights the urgency of implementing public policies that strengthen care systems. This study highlights the difficult role of informal caregivers, who are fundamental to the well-being of people with chronic illnesses and/or dependencies.

The findings highlight how family support not only acts as an essential resource for mitigating the stress associated with caregiving, but also contributes to the emotional resilience of caregivers. However, economic barriers and limited access to health services represent significant challenges that require urgent attention.

It is imperative that public policies recognize and value the work of informal caregivers, implementing strategies that improve their working conditions and promote their physical and emotional well-being. This includes the development of training programs, financial support, and the creation of support networks that mitigate overload and encourage a more equitable distribution of caregiving responsibilities. These actions are essential to guarantee dignified and sustainable care, improve the quality of life of caregivers, and promote their overall well-being in the region.

## Data Availability

The research data are available within the article.
